# Optimisation of Selective Laser Melted Ti6Al4V Functionally Graded Lattice Structures Accounting for Structural Safety

**DOI:** 10.3390/ma15249072

**Published:** 2022-12-19

**Authors:** Lei Zhu, Xiaoyang Wang, Liao Sun, Quandong Hu, Nan Li

**Affiliations:** 1 Dyson School of Design Engineering, Imperial College London, London SW7 2AZ, UK; 2 The First Aircraft Institute of AVIC, Xi’an 710087, China; 3 Manufacturing Technology Institute (MTI) of AVIC, Beijing 100024, China

**Keywords:** functionally graded lattice structure, filleted lattice metamaterials, yield stress constraint, structural optimisation, additive manufacturing

## Abstract

This paper presents a new framework for lightweight optimisation of functionally graded lattice structures (FGLSs) with a particular focus on enhancing and guaranteeing structural safety through three main contributions. Firstly, a design strategy of adding fillets to the joints of body-centred cubic (BCC) type lattice cells was proposed to improve the effective yield stress of the lattices. Secondly, effective properties of lattice metamaterials were experimentally characterised by conducting quasi-static uniaxial compression tests on selective laser melted specimens of both Ti6Al4V BCC and filleted BCC (BCC-F) lattices with different relative densities. Thirdly, a yield stress constraint for optimising FGLSs was developed based on surrogate models quantifying the relationships between the relative density and the effective properties of BCC and BCC-F lattices developed using experimental results assisted by numerical homogenisation. This framework was tested with two case studies. Results showed that structural safety with respect to avoiding yield failure of the optimised FGLSs can be ensured and the introduction of fillets can effectively improve the strength-to-weight ratio of the optimised FGLSs composed of BCC type lattices. The BCC-F FGLS achieved 14.5% improvement in weight reduction compared with BCC FGLS for the Messerschmitt-Bölkow-Blohm beam optimisation case study.

## 1. Introduction

Metamaterials are materials engineered to exhibit designed properties. Lattice metamaterials are biomimetic lightweight materials that are usually constructed by repeating networks of lattice cells [1]. The design of lattice metamaterials is often inspired by naturally lightweight hierarchical structures including bamboo stems [2,3] and human proximal femurs [4,5]. Recently, lattice metamaterials have attracted increasing attention in engineering sectors (e.g., the turbine blades design presented by Alkebsi et al. [6]) and medical applications (e.g., the hip implant structural designs presented by Gok [7]). This is owing to the advanced properties of lattice metamaterials, such as the high stiffness-to-weight ratio, high strength-to-weight ratio, excellent heat transfer capability, and outstanding energy absorption capability [8,9]. Additive manufacturing (AM), also known as 3D printing, employs a layer-wise fabrication method directly according to the data from CAD models. It provides the feasibility of fabricating structures with complex geometries, such as lattice metamaterials, that are difficult to manufacture with conventional manufacturing methods. With the rapid development recently, AM significantly increases the freedom of lightweight design and has become an important enabler of the fabrication of lattice metamaterials [10,11]. Typical AM methods for fabricating metallic lattice metamaterials include selective laser melting (SLM) [12] and direct metal laser sintering (DMLS) [13].

Experimental investigations of deformation behaviour and effective mechanical properties (e.g., effective elastic moduli and effective yield stresses) of lattice metamaterials have been conducted in a number of studies. The investigations are usually carried out by conducting quasi-static uniaxial compression tests on lattice metamaterials with various cell topologies and different parent materials (i.e., the material that lattice metamaterials are composed of) at a range of relative densities. For instance, Crupi et al. [14] investigated the effects of lattice cell size and relative density on the failure modes of a body-centred cubic (BCC) lattice made of Ti6Al4V manufactured using DMLS; Choy et al. [15] studied the effective properties and compressive deformation behaviour of a Ti6Al4V primitive cubic (PC) lattice fabricated with the SLM method; Alsalla et al. [16] adopted the microcomputer tomography method to investigate the yield and fracture behaviour of a 316 L gyroid lattice fabricated using SLM. Recently, experimental studies have been conducted to investigate effective properties of advanced lattice metamaterials, such as lattices with graded relative densities [17,18] and crystal-microstructure-inspired lattices with hybrid lattice cell types [19,20]. Yield behaviours of lattice metamaterials have been tested in these studies.

Meanwhile, lattice metamaterials have also drawn great interests from researchers on computational mechanics to develop recent advances from the structural design and optimisation perspective. This is mainly motivated by the potential of functionally graded lattice structures (FGLSs) in achieving superior functional performances, by efficiently tailoring the distributions of lattice configurations. Owing to the regular arrangement of struts in a lattice cell, the geometry of a lattice cell can be described by a limited number of geometric parameters, such as strut diameters. Thus, the effective material properties (e.g., effective elasticity tensor) of lattice metamaterials can be engineered by controlling the geometric parameters. Optimisation frameworks have been developed using such geometric parameters as design variables to obtain the optimal design of FGLSs. For example, Takezawa et al. [21] adopted the PC type lattice to design an FGLS for a liquid cooling system; Simsek et al. [22] employed the gyroid type lattice to optimise the dynamic performance of an FGLS for a desired bandgap; Wang et al. [23] proposed a two-step approach of optimising lattice structures applied in aerospace components, such as a trapezoidal rudder. Numerical homogenisation is a well-accepted and widely adopted method to characterise the effective properties of lattice metamaterials to be used in optimisation frameworks for the design of FGLSs. However, there can be a discrepancy between the numerically characterised effective properties and the actual effective properties of AM fabricated lattice metamaterials.

To bring the research on the design of FGLSs closer to applications, imposing failure constraints, such as yield stress constraints, is crucial, in terms of ensuring the structural safety. The von Mises yield criterion is widely adopted in formulating the yield stress constraint in structural optimisation, such as topology optimisation [24,25,26], and has been applied to the optimisation of FGLSs [27,28]. For instance, Zhang et al. [29] adopted the numerical homogenisation method [30] to characterise the effective elastic moduli and effective yield stresses of octet type lattices at a range of relative densities. They used the homogenised effective yield stresses to develop aggregated yield stress constraints based on the von Mises yield criterion. In their study, the design domain was divided into multiple subdomains, and the aggregation function of yield stress constraints was formulated using the averaged stresses inside each subdomain to locally control the stress levels. Thillaithevan et al. [31] developed a global yield stress constraint based on the von Mises yield criterion for optimising FGLSs. In their study, the global yield stress constraint controlled only the maximum homogenised effective von Mises stress of the lattice metamaterial to significantly reduce computational cost. Because lattice metamaterials are capable of exhibiting orthotropic material properties, the Hill’s yield criterion has also been adopted in predicting the yield stress of orthotropic lattice metamaterials [32] and in formulating stress constraints for the design of FGLSs with orthotropic lattice metamaterials. The Hill’s yield criterion is a generalised von Mises criterion for orthotropic materials [33]. Cheng et al. [34] employed the Hill’s yield criterion to develop a global yield stress constraint for the optimisation of FGLSs with a PC type lattice. In their study, the effective values of normal yield stresses, shear yield stresses, and the hydrostatic yield stress of the lattice metamaterials were characterised using a numerical homogenisation method. However, again, these studies adopted purely numerical methods without incorporating experimentally measured effective properties from actual AM fabricated lattice structures. Therefore, the structural safety may not be guaranteed for real applications.

By replacing the numerical homogenisation with the direct experimental characterisation of lattice metamaterials, the effects of manufacturing on the effective properties of lattice metamaterials can be considered, which obviously means the performance of lattice structures can be more accurately predicted in simulations, thus the optimised structure can be more reliable. However, the fact is that few of the existing studies on the optimisation of FGLSs have adopted an experimental approach to determine the effective properties of lattice metamaterials, just like research focused on experimental studies of AMed lattices has rarely applied their findings to the structural design and optimisation of the lattice structures. Therefore, to facilitate the development of FGLSs towards real applications as high value-added components, the synergy between experimental and numerical studies and a holistic methodology for designing such a new family of structures are highly desired.

In this paper, we present a new optimisation framework for numerical optimal design of high-stiffness and lightweight FGLSs, with a unique focus on improving and ensuring structural safety by innovatively introducing joint fillets to the lattice cell design for increased effective yield stress, integrating the experimental testing of lattice metamaterials as a new step for accurately characterising effective properties, and adding a yield constraint to the optimisation to avoid yield failure of the optimised FGLSs. A typical bending dominated lattice type, body-centred cubic (BCC), as well as the representative parent material and AM approach, Ti6Al4V and selective laser melting (SLM), respectively, are adopted in this study to demonstrate the framework and methodologies. The abbreviations used in this paper are tabulated in Table 1.

## 2. Materials and Methods

Due to the unconventional nature of the study and its workflow, the structure and methodology of this work is firstly overviewed in this section. Subsequently, the geometries of BCC type lattice unit cells for the lattice metamaterial were designed in this section. Fillets were introduced to the joints of lattice cells to reduce stress concentration. The effects of fillet radii on the effective yields stresses of the BCC type lattices were investigated through a parametric study using numerical homogenisation. The procedures for experimental characterisation of the effective material properties (i.e., effective values of Young’s moduli, Poisson’s ratios, and yield stresses) of the BCC and BCC-F lattices are presented in this section.

### 2.1. Overview of Workflow and Methodologies

**Figure 1:**
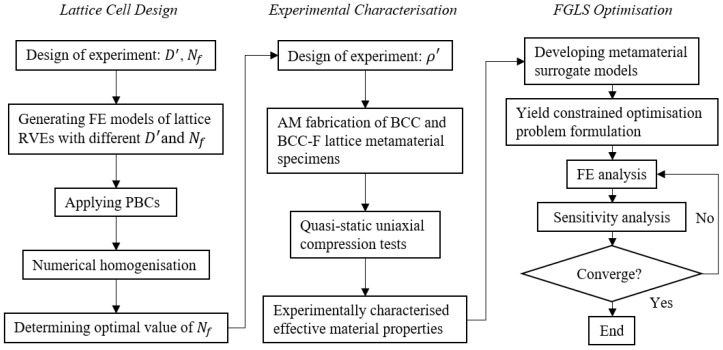
Flowchart of the yield constrained FGLS optimisation framework with experimentally characterised BCC and BCC-F lattice metamaterials. $D^{\prime }$ and $N_{f}$ denote relative strut diameter and fillet parameter, respectively, which are defined in the next subsection (through Equations (1) and (2), respectively). FE, AM, RVE, PBC, FGLS denote finite element, additive manufacturing, representative volume element, periodic boundary condition, and functionally graded lattice structure, respectively.

This subsection overviews the structure and methodologies adopted in this paper. The flowchart in Figure 1 shows the workflow of developing the experimentally characterised, yield stress constrained FGLS optimisation framework embodied by the optimal design of lattice structures composed of filleted BCC lattice cells. The workflow can be divided into three stages. (i) The first stage is the lattice cell design stage. In this stage, we improved the joint geometry design for BCC lattice cells by introducing the fillet to increase the yield stress of the BCC lattice. (For brevity, BCC-F denotes the filleted BCC lattice in this paper.) Numerical parametric studies were carried out to determine the optimal value of the fillet parameter, $N_{f}$ (given in Equation (2)). In the numerical studies, the finite element (FE) models of BCC type lattice unit cells with different strut diameters ($D^{\prime }$) and $N_{f}$ were built. The lattice unit cells were treated as representative volume elements (RVEs) of the lattice metamaterials. Numerical uniaxial loading tests were conducted on the RVEs with the periodic boundary conditions (PBCs) [35] applied to evaluate the effective yield stress of each RVE to determine the optimal value of $N_{f}$. This optimal value of $N_{f}$ was then adopted to design testing specimens for the experimental characterisation of lattice metamaterials in the second stage. (ii) The second stage is experimental characterisation. The designed specimens composed of BCC and BCC-F lattices were fabricated using SLM. Quasi-static uniaxial compression tests were conducted on these specimens to characterise their effective Young’s moduli, effective Poisson’s ratios, and effective yield stresses. (iii) In the third stage, the experimentally obtained results, combining FE analysis, were used to develop metamaterial surrogate models quantifying the relationships between the effective properties and the relative densities of BCC and BCC-F; then, these surrogate models were employed to formulate yield constrained optimisation problems for designing FGLSs. The yield stress constraint was developed based on the von Mises yield criterion. An optimisation platform integrating FE analysis and sensitivity solvers was developed to implement the optimisation of FGLSs and was tested through two case studies.

### 2.2. Unit Cell Design for Lattice Metamaterials

#### 2.2.1. Design of BCC Type Lattice Unit Cells

**Figure 2:**
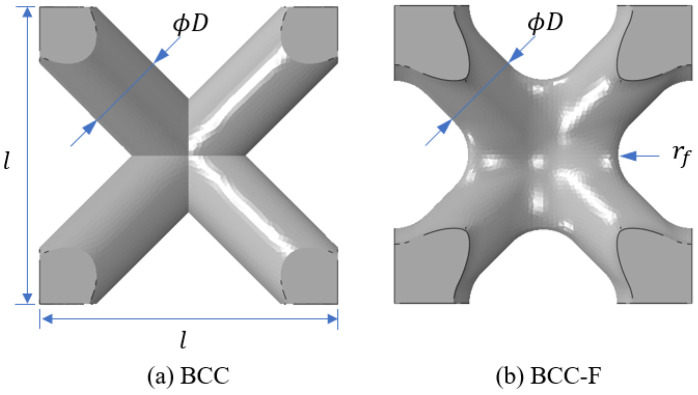
Configurations of (**a**) a BCC lattice cell and (**b**) a BCC-F lattice cell, shown in the front view, where l, D, and $r_{f}$ denote the length of the lattice cell, the lattice strut diameter, and the fillet radius, respectively.

The BCC lattice cell type is a bending dominated lattice type [1,36,37] and yield is a common failure mode of this lattice type. Thus, this lattice cell type was selected in this study to demonstrate the development of the yield constrained lattice structural optimisation framework. The lattice struts are usually connected by simple Boolean operations during the construction of lattice CAD models, resulting in sharp conners in joint regions of the lattice cells where the lattice struts are intersected [38]. These sharp conners can result in severe stress concentration in the joint region of lattice cells, resulting in premature yield failure of the lattice cell. To tackle the problem, in this study, a fillet design was introduced to the joints of lattice cells to smooth the joint conners. The comparison of an original BCC lattice cell and a filleted BCC (BCC-F) lattice cell is demonstrated in Figure 2, where l is the length of a lattice cell, D is the diameter of a lattice sturt, and $r_{f}$ is radius of a fillet. In this study, for each BBC-F lattice cell, the diameters of all struts were defined as equal, and all joint corners shared the same radius.

Adding fillets to lattice joints results in adding extra material to lattice cells. The admissible space in a lattice cell for adding fillets is restricted by the topology and the strut dimeter of the lattice cell. In general, a lattice cell with a higher strut diameter leaves a smaller space for adding fillets to its joint. Thus, in this study, the assumption was made that the strut diameter, D, and the fillet radius, $r_{f}$, generally had a multiplicative inverse relationship. For the convenience of defining the relationship between D and $r_{f}$, the dimensionless relative strut diameter, $D^{\prime }$, and relative fillet radius, ${r^{\prime }}_{f}$, can be defined as:

(1) $$\;D^{\prime }=D/l,\;\;{r^{\prime }}_{f}=r_{f}/l$$

In this study, the reciprocal relationship between $D^{\prime }$ and ${r^{\prime }}_{f}$ can be defined as:

(2) $$\;\;\;\;\;{r^{\prime }}_{f}=N_{f}/\left(250D^{\prime }\right)$$

where $N_{f}$ is a fillet parameter that controls the fillet size relative to the strut diameter.

#### 2.2.2. Numerical Characterisation of Lattice Metamaterials and Parametric Study on the Fillet Design

**Figure 3:**
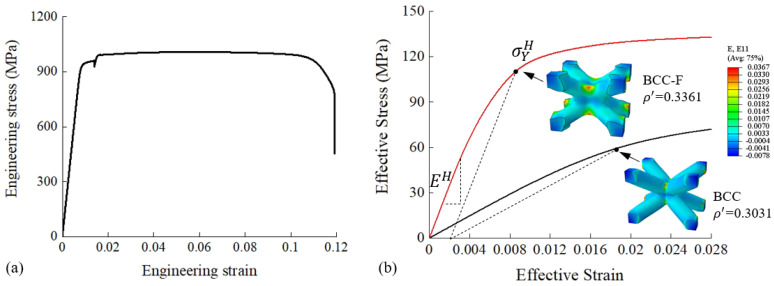
The stress–strain curves of (**a**) parent material, i.e., SLM fabricated Ti6Al4V, for lattice metamaterials, and (**b**) examples of a BCC-F and a BCC lattice RVEs, where $E^{H}$ and $\sigma_{Y}^{H}$ are the effective Young’s modulus and the effective yield stress, respectively. The stress–strain curves of the parent material and the example RVEs were obtained experimentally and numerically, respectively.

The effect of $N_{f}$ on the effective yield stress of the BCC type lattices was investigated in a numerical parametric study. A lattice cell with larger $N_{f}$ requires the adding of more material to the joint region, which results in a smaller lattice strut diameter to maintain the same level of relative density. The minimum value of the strut diameter is restricted by the manufacturing capability, which in turn restricts the maximum value of $N_{f}$. In this study, the maximum value of $N_{f}$ was determined to be 10 so that the minimum strut diameter would not exceed the manufacturing requirement. The parametric study was conducted numerically using Abaqus 2018. As previously mentioned, lattice metamaterials are composed of periodically distributed lattice unit cells, which can be treated as RVEs. Numerical homogenisation was conducted on the RVEs of BCC and BCC-F lattices with different $N_{f}$ values at three relative density levels, i.e., $\rho^{\prime }=0.1,\;0.3,\;0.5$. The value of $N_{f}$ was specified to range from 1 to 10. Periodic boundary conditions (PBCs) were applied to each RVE by using EasyPBC [35] in Abaqus. The tetrahedral C3D4 element type was adopted. The mesh size was determined through a mesh size convergence study to balance accuracy and efficiency. The parent material of the lattice metamaterials was set to be selective laser melting (SLM) fabricated Ti6Al4V and its properties were obtained by conducting quasi-static uniaxial tensile tests on standard tensile specimens of Ti6Al4V fabricated using SLM. The stress–strain curve of the parent material is shown in Figure 3a. The Young’s modulus was 117.5 GPa, the Poisson’s ratio was 0.34, and the yield stress (0.2% offset) was 939.625 MPa. Using the experimentally measured parent material properties, the effective mechanical properties of the lattice metamaterials could be computed. The 0.2% offset yield stress was used to determine the effective yield stresses of the lattice metamaterials. Figure 3b illustrates examples of the effective stress-effective strain curves of a BCC RVE, and a BCC-F RVE obtained from the numerical parametric study based on varied $\rho^{\prime }$ and $N_{f}$, where the determination of effective Young’s modulus ($E^{H}$) and effective yield stress ($\sigma_{Y}^{H}$) is demonstrated.

**Figure 4:**
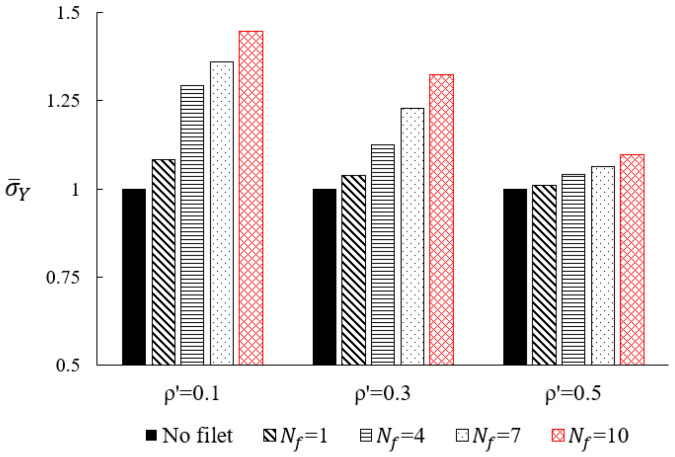
Effect of the fillet parameter, $N_{f}$, on the normalised effective yield stress, ${\bar{\sigma }}_{Y}$, of the BCC type lattice at different relative density ($\rho^{\prime }$ ) levels. At each $\rho^{\prime }$ level, the yield stress values are normalised by the yield stress of the BCC lattice without fillets.

To determine the optimal value of $N_{f}$, the effects of $N_{f}$ on the effective yield stress of the BCC type lattice metamaterial at different relative density levels are shown in Figure 4. At each relative density level, the yield stress of each case was normalised by the effective yield stress of the BCC lattice without fillets. The normalised yield stress monotonically increased with the increase of $N_{f}$ at each relative density level, indicating that the effective yield stress of the BCC type lattice can be improved by increasing the fillet radius in the given range. Although this improvement decreased with the increase in relative density, the lattice metamaterial with $N_{f}$ = 10 showed the highest normalised yield stress at each relative density level. Thus, $N_{f}$ = 10 was determined to be the optimal value for the fillet design in this study and would be adopted in the specimen designs and experimental characterisation of the BCC-F lattice in the following section.

### 2.3. Experimental Characterisation of BCC and BCC-F Lattice Metamaterials

#### 2.3.1. Design and Fabrication of the BCC and BCC-F Lattice Specimens

It has been discussed in Section 2.2.2 that the effective yield stress of the BCC type lattice increases monotonically with the increase of the fillet parameter, $N_{f}$, within the given range of $N_{f}$. To reduce the number of tests, two groups of specimens, the BCC lattice (without fillets) and the BCC-F lattice with the maximum $N_{f}$, i.e., $N_{f}$ = 10, were designed to be fabricated for uniaxial compression tests characterising the effective material properties of the lattice metamaterials. Each specimen group contained three lattice metamaterial specimen designs with relative strut diameters, $D_{ds}^{\prime }$, of 0.144, 0.270, and 0.376, respectively.

**Figure 5:**
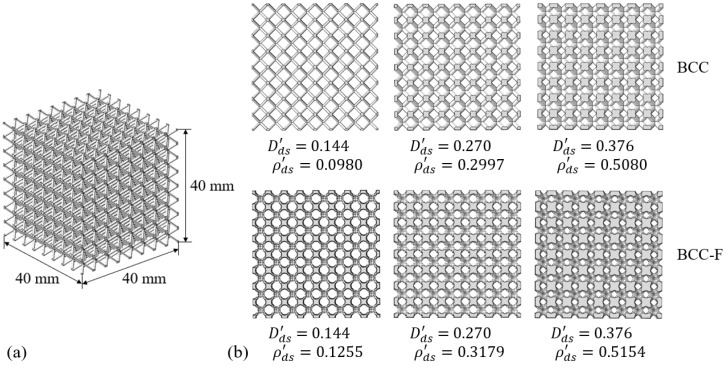
Demonstration of (**a**) dimensions of lattice specimen design and (**b**) CAD models (front view) of BCC and BCC-F specimen designs, where $D_{ds}^{\prime }$ and $\rho_{ds}^{\prime }$ represent designed values of relative strut diameter and relative density, respectively.

Different from the numerical homogenisation method with PBC applied to a single lattice unit cell, the direct experimental characterisation was conducted on actual lattice structures, architected by a sufficient amount of repeatedly arranged identical lattice cells for each lattice design configuration. Each lattice metamaterial specimen was designed to be a cubic containing 512 (8 × 8 × 8) lattice cells with a uniform cell dimension of 5 × 5 × 5 mm, resulting in the total dimension of a lattice metamaterial specimen being 40 × 40 × 40 mm. The CAD models of specimen designs of the BCC and BCC-F lattices with different designed $D_{ds}^{\prime }$ and corresponding relative densities, $\rho_{ds}^{\prime }$, are demonstrated in Figure 5.

The specimens were fabricated by using SLM with the same parent material, Ti6Al4V, as given in Section 2.2.2. The processing parameters of SLM are tabulated in Table 2. Three duplicates were fabricated for each specimen design. All the specimens were separated from the support material by wire cutting after fabrication. Annealing heat treatments were performed on the specimens at 800 °C for 4 h.

**Figure 6:**
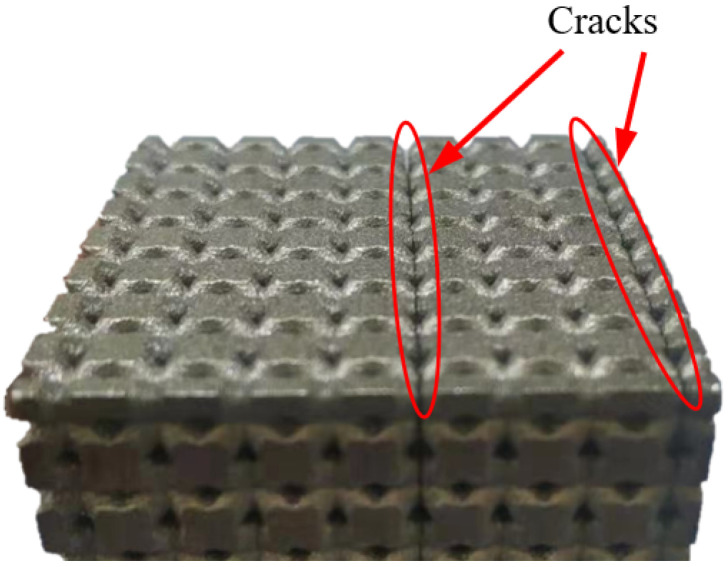
Cracks observed on a high relative density ($\rho_{ds}^{\prime }=0.7$) BCC lattice specimen after fabrication.

The reason for designing the maximum relative density of the lattice metamaterials to be 0.51 in this study is briefly explained as follows: it was observed that specimens with a higher relative density fractured after fabrication. Figure 6 shows the cracks on a BCC lattice sample with a relative density of 0.7. The cracks could be caused by the high residual stress generated in the lattice specimen during the SLM manufacturing process. The increase in strut diameter could cause the rise of the temperature gradient in the cross section of lattice struts, consequently resulting in higher residual stresses. Cracks could be generated when the residual stresses exceed the tensile stress of the printed parent material.

**Figure 7:**
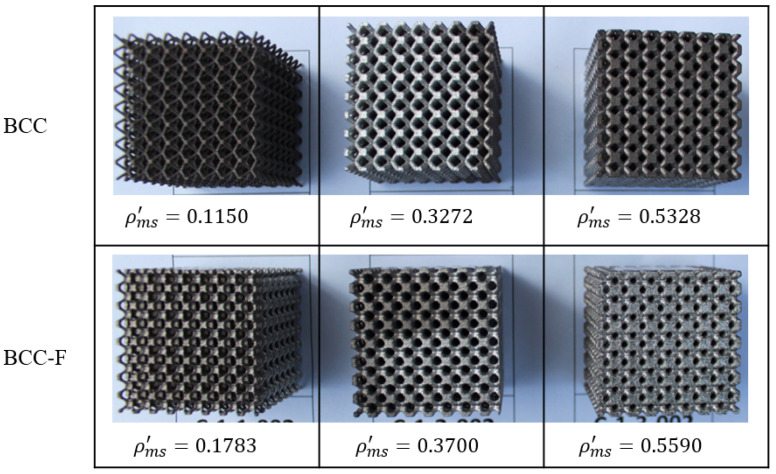
Demonstration of SLM fabricated BCC and BCC-F lattice specimens with different measured relative densities ($\rho_{ms}^{\prime }$).

The two fabricated groups of specimens with different measured relative densities, $\rho_{ms}^{\prime }$, are demonstrated in Figure 7. The measured relative density values were higher than the designed ones for all specimens, which can be caused by manufacturing errors and defects, such as adhesion of additional powders on the surfaces of lattice struts [15,39,40].

#### 2.3.2. Experimental Setup and Procedure of Uniaxial Compression Tests

Uniaxial compression tests were conducted to characterise the effective mechanical properties of the fabricated BCC and BCC-F lattices. The effective mechanical properties to be experimentally measured include the effective Young’s modulus, the effective Poisson’s ratio, and the effective yield stress.

**Figure 8:**
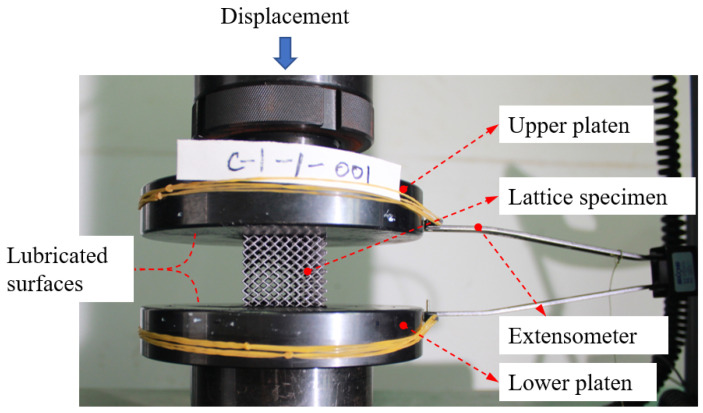
Experimental setup of the quasi-static uniaxial compression tests on the specimens constructed by BCC and BCC-F lattices at room temperature.

The experimental setup is shown in Figure 8. The experiments were conducted on an MTS E64 machine, following the ISO 13314:2011 standard of compression test for porous and cellular metals [41]. The lattice metamaterial specimen was placed between upper and lower 42CrMo4 steel (52 HRC) platen grips. The contacting interfaces between the specimen and the platen grips were lubricated with MoS2 lubricant to reduce the friction when the specimen expanded off the loading direction under compression. During a test, the compressive load was measured using a load cell and the displacement (along the loading direction) of the specimens was measured using an extensometer attached to the platen grips. The experiments were conducted at room temperature with a loading rate of 2 mm/min.

## 3. Results and Discussion

The experimental results of the uniaxial compression tests on BCC and BCC-F lattices are presented and discussed in this section. Based on the experimental results, the effective material properties (i.e., effective values of Young’s moduli, Poisson’s ratio, and yield stress) of BCC and BCC-F lattices were characterised and evaluated. Subsequently, surrogate models of both lattices were developed by adopting the experimental characterisation results with the assistance of numerical homogenisation simulations to quantify the relationship between relative density and effective material properties of the lattices. The surrogate models were then used to develop a yield stress constrained optimisation framework for the design of functionally graded lattice structures. The framework was tested with an L-shaped beam bending case study and a Messerschmitt-Bölkow-Blohm (MBB) beam bending case study.

### 3.1. Experimental Results and Discussion of The Compression Tests

**Figure 9:**
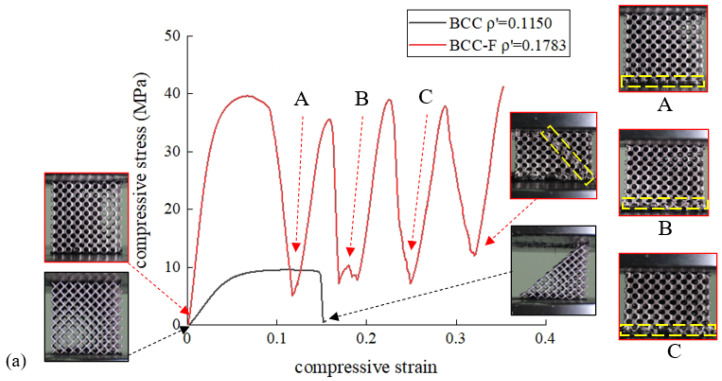

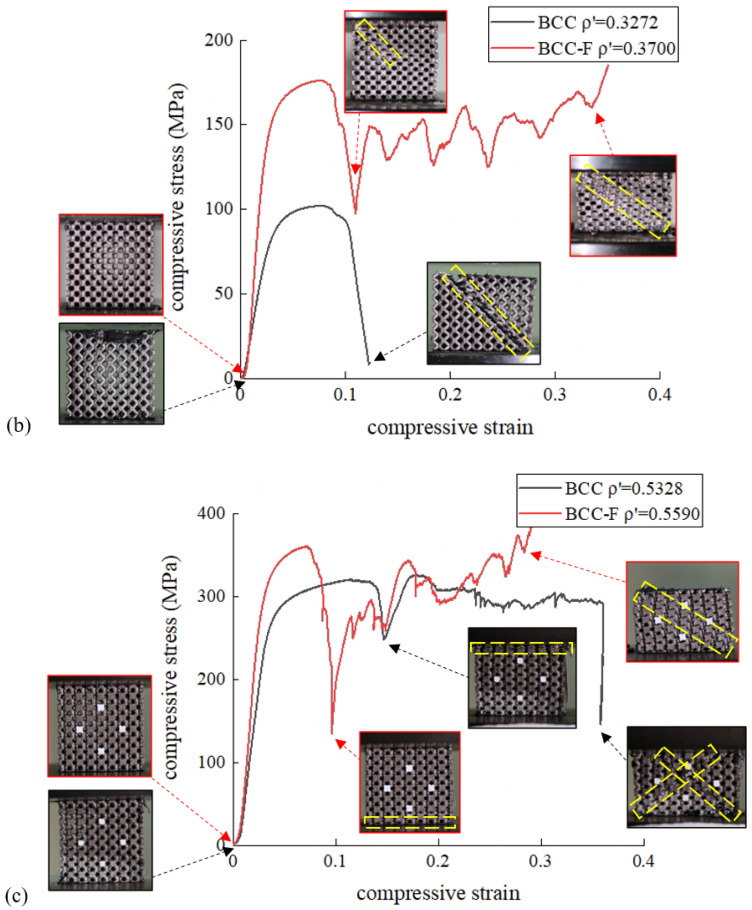
Experimental results of the compressive stress–strain (engineering) curves of specimens of (**a**) BCC lattice with $\rho^{\prime }$ = 0.1150, BCC-F lattice with $\rho^{\prime }$ = 0.1783, (**b**) BCC lattice with $\rho^{\prime }$ = 0.3272, BCC-F lattice with $\rho^{\prime }$ = 0.3700, and (**c**) BCC lattice with $\rho^{\prime }$ = 0.5328, BCC-F lattice with $\rho^{\prime }$ = 0.5590, associated with demonstrations of deformation processes and fracture modes.

All the experimental results are presented based on the measured relative density values in this section, hence, for the purpose of brevity the relative density refers to the experimentally measured relative density in the following contents of this section if not annotated. The experimental results of the engineering stress–strain curves of the BCC and the BCC-F specimens at different relative density levels are shown in Figure 9. For all specimens, the stresses increased linearly during elastic deformation until the specimens yielded and continued to increase nonlinearly toward the first peak until an abrupt drop occurred. For the BCC specimen (composed of non-filleted lattices) with a low relative density ($\rho^{\prime }$ = 0.1150 and $\rho^{\prime }$ = 0.3727), a 45° shear band propagated diagonally throughout an entire specimen, causing the fracture failure of the whole specimen after a single stress peak. A similar fracture failure mode of a low relative density Ti6Al4V BCC lattice was observed in the study of [42]. When the relative density of the BCC specimen increased to 0.5328, the first abrupt drop in stress occurred due to the fracture of the top layer of lattice cells, followed by plateau stress fluctuation attributed to the gradual propagation of the diagonal shear bands, which eventually developed into X-shaped shear bands.

For the BCC-F specimens (composed of filleted lattices), when the relative density $\rho^{\prime }$ = 0.1783, the specimen collapsed layer by layer after the first stress peak, resulting in stress fluctuation in the plateau stress region, until a diagonal shear band was propagated throughout the specimen, ending with specimen densification. The layer-by-layer collapse mode could be attributed to the geometrical nonuniformity of lattice struts caused by manufacturing errors. This geometrical nonuniformity could result in stress localisation in a lattice layer, and consequently cause premature collapse of this lattice layer. This layer-by-layer collapse mode of the BCC lattice was also observed in the study of a gradient BCC lattice in [42], where the layer with thinner strut collapsed first. When the relative density of the BCC-F lattice increased to 0.3700, the compressive stress abruptly dropped after the first stress peak, resulting from the formation of a localised diagonal shear band on the top left conner of the specimen. The localised shear band was then propagated throughout the specimen, resulting in the stress fluctuation during the densification of the specimen. For the BCC-F lattice specimen with $\rho^{\prime }$ = 0.5590, the stress abruptly dropped after the first stress peak due to the collapse of the bottom layer of lattice cells; then, the stress fluctuated, caused by the propagation of the diagonal shear band during the specimen densification stage. Similar to the conclusion drawn by Choy et al. [18], with the increase in the relative density, the deformation behaviour of the BCC and BCC-F lattices exhibited a tendency towards the deformation behaviour of a solid block.

Because the aim of the uniaxial compression tests was to characterise the effective material properties of the lattice metamaterials for the purpose of developing surrogate models used in the lattice structural optimisation, a detailed discussion on the fracture modes of lattice metamaterials is out of the scope of this research. This study focuses on the elastic regions and the yield stresses of the stress–strain curves. Good repeatability of the stress–strain curves before yield was observed from repeated tests (on three duplicated specimens). The effective Young’s moduli, effective Poisson’s ratios, and effective yield stresses of the BCC and BCC-F lattices with different relative densities will be evaluated and compared in the next subsection.

### 3.2. Evaluation of Effective Properties

The effective Young’s moduli were obtained by measuring the slopes of the stress–strain curves in the elastic deformation domains. The effective yield stresses were obtained by measuring the 0.2% offset stresses of the stress–strain curves. The effective Poisson’s ratios were obtained by measuring the vertical compressive strains and the horizontal expansion strains in the elastic deformation domains, where the strains were measured by adopting the digital image correlation method.

**Figure 10:**
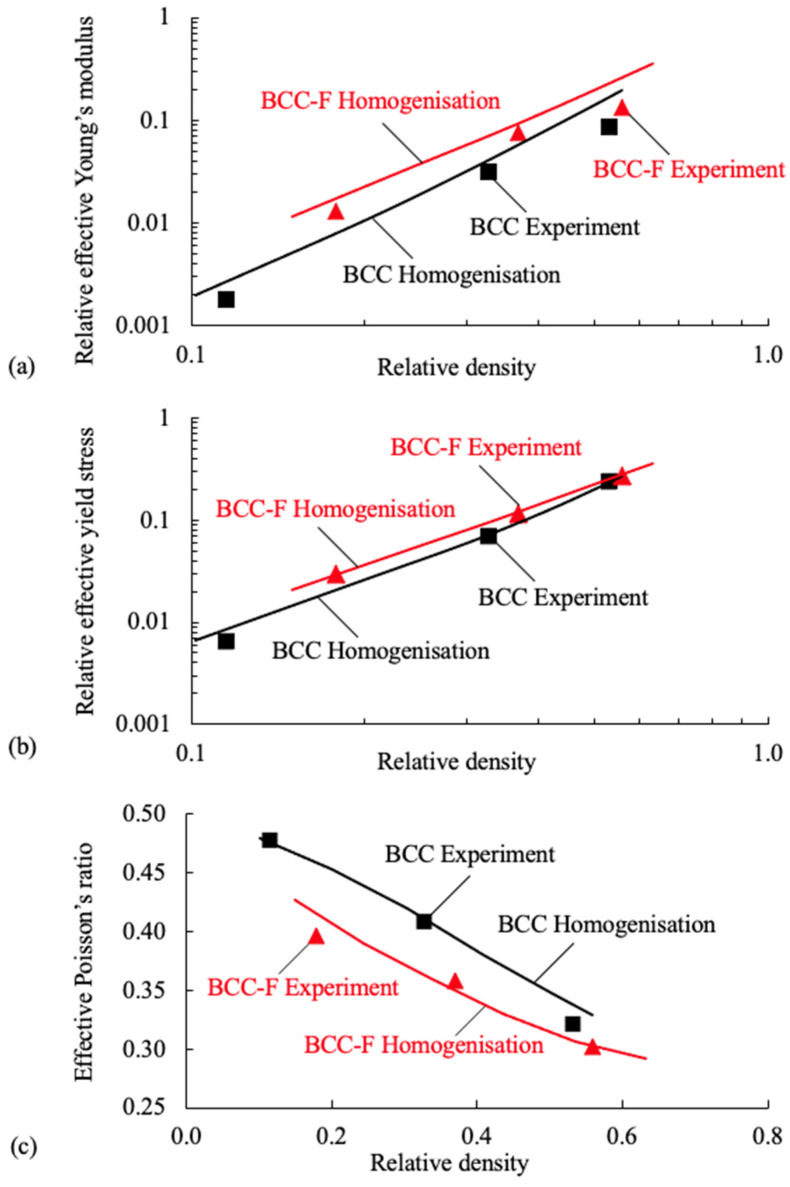
Experimental (symbols) and numerical homogenisation (solid lines) results of (**a**) the relative effective Young’s moduli (log-log scale), (**b**) the relative effective yield stresses (log–log scale), and (**c**) the effective Poisson’s ratios (linear scale) of the BCC and the BCC-F lattices at different relative density levels.

Both the experimental and numerical homogenisation results of the relative effective Young’s modulus, the relative effective yield stress, and the effective Poisson’s ratio of the BCC and the BCC-F lattices are shown in Figure 10. For the convenience of comparison, the effective Young’s moduli and yield stresses were normalised by the Young’s modulus and the yield stress of the parent material, respectively. It can be observed from Figure 10a–c that the relative effective Young’s moduli and relative effective yield stresses of both BCC and BCC-F lattices increase with the increasing relative density, while the effective Poisson’s ratios exhibit an opposite trend, for both the experimental and numerical homogenisation results.

For the experimental results (symbols in Figure 10), Figure 10a shows that the experimental relative effective Young’s moduli of the BCC-F lattices are higher than those of the BCC lattices at the same relative density level, indicating that adding fillets to the joints of the BCC lattice cells can contribute to the improvement of the effective Young’s moduli of the lattice metamaterials. This phenomenon can be attributed to the fact that the BCC lattice type is bending dominant, thus, when the lattice cells are subject to load along normal directions, the highest stress appears at the roots of lattice struts, i.e., at the joint of lattice cells. Hence, adding fillet can increase the amount of material in the high stress region, and consequently provide higher stress resistance. Figure 10b shows that the experimental relative effective yield stresses of the BCC-F lattices are higher than those of the BCC lattices at each relative density level, indicating that adding fillets to the joints of the BCC lattice cells can reduce the stress concentration in the lattice joints, consequently improving the yield stresses of the lattice metamaterials. This improvement decreases with the increase in relative density, as the result of the reciprocal relationship between the strut diameter and fillet radius of the lattice cells proposed in this study, recall Section 2.2.2. As can be observed in Figure 10c, the effective Poisson’s ratios of the BCC lattice are higher than those of the BCC-F lattice at the same relative density level.

To compare the numerical homogenisation results against the experiment results, Figure 10 shows that the numerical homogenisation results can predict the trends of all properties for both BCC and BCC-F lattices correctly, but cannot predict the values of all such properties accurately. Figure 10a shows that the numerical homogenisation results (solid lines) of the relative effective Young’s moduli are higher than the experimental results for both BCC and BCC-F lattices. Figure 10b shows that the errors between the simulation results and the experimental results of the relative effective yield stresses are relatively small for lattices with high relative densities, however, the simulation results are higher than the experimental results for lattices with small relative densities. Figure 10c shows that there are discrepancies between the simulation and experimental results of the effective Poisson’s ratios of both BCC and BCC-F lattices. The discrepancy between the effective material properties of lattice metamaterials characterised numerically and experimentally could be attributed to the effects of manufacturing defects and errors, such as the waviness and shape irregularity of lattice struts, on the mechanical properties of the lattice metamaterials not being considered when constructing the FE models for the simulations. Thus, it emphasised the necessity of using experimental results for characterising the effective material properties of lattice metamaterials.

### 3.3. Development of a Yield Constrained Optimisation Framework for Functionally Graded Lattice Structural Designs

#### 3.3.1. Development of Surrogate Models of BCC and BCC-F Lattices

The surrogate models of effective properties of the BCC and BCC-F lattices are developed in this subsection. The models for the effective elasticity tensor components related to Young’s moduli and Poisson’s ratios, and for effective yield stresses, were developed by adopting the experimental data obtained in Section 3.1. The shear moduli were required for the development of surrogate models of the effective elasticity tensors. However, shear tests were not conducted in this study due to the lack of standard for the shear test for porous and cellular metals and the restriction of testing equipment. Thus, in this study, the simulation results of shear moduli were corrected to be used for surrogate model development. The shear moduli were corrected based on the assumptions of (i) the effects of manufacturing defects on the Young’s moduli and the shear moduli of the BCC type of lattices sharing the same trend and (ii) at the same relative density, the relative error, e, between the simulation results and the experimental results being approximately equal for the Young’s moduli and the shear moduli. These assumptions were made based on the following fact: although at the higher hierarchical level, the lattice metamaterials were subject to shear stress and normal stress under pure shear tests and uniaxial compression tests, respectively, the lattice cells at the lower hierarchical level were subject to resultant forces of axial forces and bending forces under both pure shear tests and uniaxial compression tests. The relative error, e, can be calculated as:

(3) $$\;e=\left(E^{H}-E^{E}\right)/E^{H}$$

where $E^{H}$ and $E^{E}$ represent the numerically homogenised effective Young’s modulus and the effective Young’s modulus measured from experiments, respectively. The relative error was calculated at each relative density level for both the BCC and the BCC-F lattices. The relationship between the relative error, e, and the relative density, $\rho^{\prime }$, can be obtained through regression using second-order polynomial functions shown in Equations (4a) and (4b), for the BCC and the BCC-F lattices, respectively:

(4a) $$\;e_{BCC}\left(\rho^{\prime }\right)=4.5888{\left(\rho^{\prime }\right)}^{2}-2.6040\rho^{\prime }+0.5731$$

(4b) $$e_{BCC-F}\left(\rho^{\prime }\right)=5.3687{\left(\rho^{\prime }\right)}^{2}-3.4265\rho^{\prime }+0.7154$$

**Figure 11:**
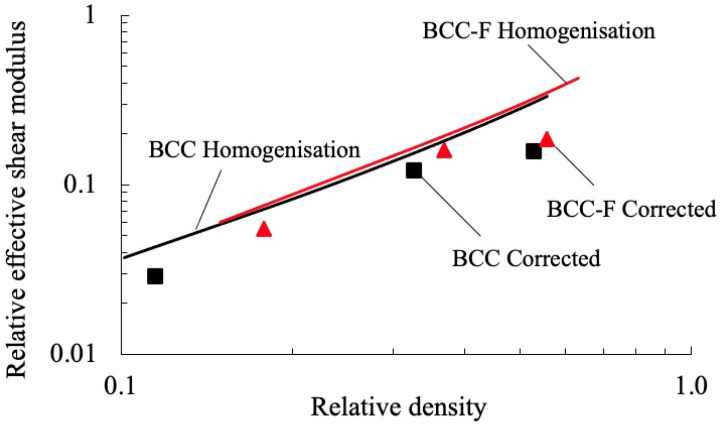
The corrected relative effective shear moduli and the corresponding numerically homogenised relative effective shear moduli of the BCC lattice and the BCC-F lattices at different relative densities (log–log scale).

The corrected values of the effective shear moduli, $G^{C}$, at a given relative density $\rho^{\prime }*$, can be calculated as:

(5) $$G^{C}{|}_{\rho^{\prime }=\rho^{\prime }*}={\left.G^{H}\cdot \left(1-e\left(\rho^{\prime }\right)\right)\right|}_{\rho^{\prime }=\rho^{\prime }*}$$

where $G^{H}$ is the numerically homogenised effective shear modulus. In Figure 11, the corrected values of the relative effective shear moduli (symbols) for the BCC and the BCC-F lattices were calculated at each relative density and plotted with the corresponding numerically homogenised effective shear moduli (solid lines), where all relative values of the moduli were calculated by normalising the effective shear moduli with the shear modulus of the parent material.

Both BCC and BCC-F lattice metamaterials can be simplified as quasi-isotropic metamaterials because the parent material was approximately isotropic. The effective elasticity tensor, $C^{E}$, of the BCC and the BCC-F quasi-isotropic lattice metamaterials can be written as:

(6) $$C^{E}=\left[\begin{array}{cccccc} C_{11}^{E} & C_{12}^{E} & C_{12}^{E} & 0 & 0 & 0\\ & C_{11}^{E} & C_{12}^{E} & 0 & 0 & 0\\ & & C_{11}^{E} & 0 & 0 & 0\\ & & & C_{44}^{E} & 0 & 0\\ & \mathrm{sym} & & & C_{44}^{E} & 0\\ & & & & & C_{44}^{E} \end{array}\right]$$

The components of the effective elasticity tensor can be calculated with the experimentally measured Young’s moduli, $E^{E}$, Poisson’s ratio, $\nu^{E}$, and the simulation-corrected shear moduli, $G^{C}$, by using Equations (7a)–(7c):

(7a) $$\;\;\;C_{11}^{E}=E^{E}\frac{1-\nu^{E}}{\left(1-2\nu^{E}\right)\left(1+\nu^{E}\right)}$$

(7b) $$\;\;\;C_{12}^{E}=E^{E}\frac{\nu^{E}}{\left(1-2\nu^{E}\right)\left(1+\nu^{E}\right)}$$

(7c) $$C_{44}^{E}=G^{C}$$

**Figure 12:**
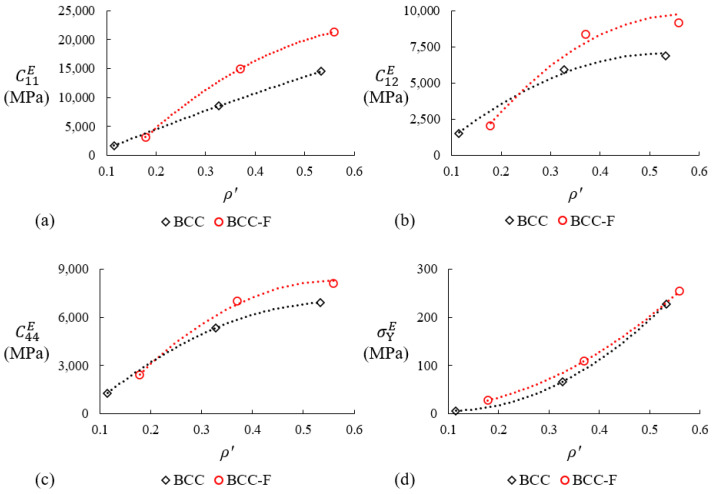
Relationship between the relative density, $\rho^{\prime }$, and the effective material properties of (**a**) $C_{11}^{E}$, (**b**) $C_{12}^{E}$, (**c**) $C_{44}^{E}$, and (**d**) 0.2% offset yield stress, $\sigma_{Y}^{E}$, of the BCC and the BCC-F lattices. Symbols represent the properties measured and calculated at specific density levels and dashed lines represent the plots of second-order polynomial surrogate models fitting the symbols.

The effective elasticity tensors of the BCC and the BCC-F lattices were calculated at each relative density level, $\rho^{\prime }$. The relationship between $\rho^{\prime }$ and $C_{11}^{E}$, $C_{12}^{E}$, $C_{44}^{E}$, and $\sigma_{Y}^{E}$, as shown in Figure 12, can be quantified by second-order polynomial surrogate models, which can be written as:

(8a) $$\;C_{ij}^{E}\left(\rho^{\prime }\right)=A_{2}^{ij}{\left(\rho^{\prime }\right)}^{2}+A_{1}^{ij}\rho^{\prime }+A_{0}^{ij}$$

(8b) $$\sigma_{Y}^{E}\left(\rho^{\prime }\right)=B_{2}{\left(\rho^{\prime }\right)}^{2}+B_{1}\rho^{\prime }+B_{0}$$

where $A_{0}^{ij}$, $A_{1}^{ij}$, and $A_{2}^{ij}$ are constants for the surrogate model of the effective elasticity component $C_{ij}^{E}$, $B_{0}$, $B_{1}$, and $B_{2}$ are constants for the surrogate model of the effective yield stress, $\sigma_{Y}^{E}$. Note that these surrogate models are only guaranteed to be valid when the value of the relative density is within the relative density range of the experimental data and the accuracy of extrapolation beyond the experimentally measured ranges is not guaranteed.

#### 3.3.2. Optimisation Problem Formulation

The yield stress constraint for the lattice metamaterials was developed based on the von Mises yield criterion. The widely used von Mises yield criterion has been adopted to formulate yield stress constraints in mono-scale topology optimisation [43,44,45] and in functionally graded lattice structural optimisation [31]. The von Mises stress, $\sigma_{VM}$, can be calculated as:

(9) $$\;\sigma_{VM}=(0.5{\left({\left(\sigma_{1}-\sigma_{2}\right)}^{2}+{\left(\sigma_{2}-\sigma_{3}\right)}^{2}+\left(\sigma_{3}-\sigma_{1}{)}^{2}\right)+3\left(\sigma_{4}^{2}+\sigma_{5}^{2}+\sigma_{6}^{2}\right)\right)}^{0.5}$$

where $\sigma_{1}$ to $\sigma_{6}$ are components of the stress tensor in Voigt notation. According to the von Mises yield criterion, material starts yielding when $\sigma_{VM}$ exceeds the yield stress of the material. To ensure the von Mises stress of each lattice cell in a lattice structure is under the corresponding yield stress, the yield constraint can be written as:

(10) $$\;\sigma_{VM}^{\zeta }\left({\rho^{\prime }}_{\zeta }\right)/\sigma_{Y}^{E\zeta }\left({\rho^{\prime }}_{\zeta }\right)-c_{s}\leq 0$$

where $\sigma_{VM}^{\zeta }\left({\rho^{\prime }}_{\zeta }\right)$ and $\sigma_{Y}^{E\zeta }\left({\rho^{\prime }}_{\zeta }\right)$ represent the von Mises stress and the yield stress of the ζth lattice cell in a lattice structure, respectively; $c_{s}$ is a safety factor equal to or smaller than one. Equation (10) constrains the stress of each lattice cell individually. Hence, the number of yield stress constraints equals the total number of lattice cells in a lattice structure. To improve computational efficiency, the yield stress constraint was only applied to the maximum value of $\sigma_{VM}^{\zeta }\left({\rho^{\prime }}_{\zeta }\right)/\sigma_{Y}^{E\zeta }\left({\rho^{\prime }}_{\zeta }\right)$ in this study. The adjusted yield stress constraint can be written as:

(11) $$\;\;\;\max\left(\sigma_{VM}^{\zeta }\left({\rho^{\prime }}_{\zeta }\right)/\sigma_{Y}^{E\zeta }\left({\rho^{\prime }}_{\zeta }\right)\right)-c_{s}\leq 0$$

Two optimisation problems, ${\mathbb{P}}_{A}$ and ${\mathbb{P}}_{B}$, were formulated in this study. Problem ${\mathbb{P}}_{A}$ was a structural compliance minimisation problem under two inequality constraints, being the maximum volume fraction constraint and the yield stress constraint. ${\mathbb{P}}_{A}$ was used to compare the von Mises stress of the BCC-F lattice structures with and without yield stress constraint. The formulation of ${\mathbb{P}}_{A}$ can be written as:

(12) $${\mathbb{P}}_{A}\left\{\begin{array}{l} \;\underset{{\rho^{\prime }}_{\zeta }}{\min}\;f\left({\rho^{\prime }}_{\zeta }\right)=0.5\;F^{T}U,\;\;\zeta =1,\ldots ,N\\ \;s.t.\;\;h\left({\rho^{\prime }}_{\zeta }\right)=K\left({\rho^{\prime }}_{\zeta }\right)U-F=0\\ \;\;g_{1}\left({\rho^{\prime }}_{\zeta }\right)=\frac{1}{V}\sum_{\zeta =1}^{N}{\rho^{\prime }}_{\zeta }V_{\zeta }-v_{f}\leq 0\\ \;\;g_{2}\left({\rho^{\prime }}_{\zeta }\right)=\max\left(\sigma_{VM}^{\zeta }\left({\rho^{\prime }}_{\zeta }\right)/\sigma_{Y}^{E\zeta }\left({\rho^{\prime }}_{\zeta }\right)\right)-c_{s}\leq 0\\ \;\;{\rho^{\prime }}_{min}\leq {\rho^{\prime }}_{\zeta }\leq {\rho^{\prime }}_{max} \end{array}\right.$$

where the design variables, ${\rho^{\prime }}_{\zeta }$, are the relative densities of lattice cells in the lattice structure; ζ and N represent the identifier and the total number of lattice cells, respectively; $V_{\zeta }$ and V denote the volume of the ζth lattice cell and the entire lattice structure, respectively; $h\left({\rho^{\prime }}_{\zeta }\right)$ is the equilibrium equality constraint; $K\left({\rho^{\prime }}_{\zeta }\right)$ is the global stiffness matrix; U and F denote the global displacement tensor and the global external force tensor; $g_{1}\left({\rho^{\prime }}_{\zeta }\right)$ is the maximum volume fraction constraint, where $v_{f}$ is the maximum volume fraction; $g_{2}\left({\rho^{\prime }}_{\zeta }\right)$ is the yield stress inequality constraint defined in Equation (11); ${\rho^{\prime }}_{min}$ and ${\rho^{\prime }}_{max}$ are the lower and the upper bounds of the design variables, ${\rho^{\prime }}_{\zeta }$. The global stiffness matrix in ${\mathbb{P}}_{A}$ can be written as:

(13) $$K\left({\rho^{\prime }}_{\zeta }\right)=\overset{N}{\underset{\zeta =1}{\sum }}Ce_{\zeta }^{T}\left(\int_{V_{\zeta }}^{}B_{\zeta }^{T}C_{\zeta }^{E}\left({\rho^{\prime }}_{\zeta }\right)B_{\zeta }dV_{\zeta }\right)Ce_{\zeta }$$

where $Ce_{\zeta }^{T}$ is the connection matrix that maps the elemental stiffness matrix to the global stiffness matrix, $C_{\zeta }^{E}$ is the effective elasticity tensor, and $B_{\zeta }$ is the elemental strain displacement tensor.

The equilibrium function in Equation (12) was solved using the finite element (FE) method. Similar to many FE-based structural optimisation frameworks, a checkerboard-like pattern of alternating material and void can be easily generated in optimised structures [46]. This checkerboard issue can be resolved by adopting filtering techniques [46]. In this study, a design variable filter was adopted to prevent the checkerboard issue during optimisation. The filtered relative density, ${\tilde{\rho^{\prime }}}_{\zeta }$, can be calculated as:

(14) $$\;\;\;{\tilde{\rho^{\prime }}}_{\zeta }\left({\rho^{\prime }}_{\gamma }\right)=\frac{\sum_{\gamma \in N_{\zeta }}H_{\gamma \zeta }{\rho^{\prime }}_{\gamma }}{\sum_{\gamma \in N_{\zeta }}H_{\gamma \zeta }}$$

where $N_{\zeta }$ is a set of lattice cells in which the central distance between the γth and the ζth lattice cells is smaller than a filter radius, $R_{min}$; $H_{\gamma \zeta }$ is a weighting function that can be written as:

(15) $$H_{\gamma \zeta }=\max\left(0,\;R_{min}-\Delta_{\gamma \zeta }\right)$$

where $\Delta_{\gamma \zeta }$ represents the central distance between the γth and the ζth lattice cells.

The upper bound, ${\rho^{\prime }}_{max}$, and the lower bound, ${\rho^{\prime }}_{min}$, of the design variables were specified to be 0.5328 and 0.1783, respectively, based on the range of experimental data, as explained in Section 2.3. To enable topology changes of higher hierarchical level structures for further structural efficiency improvement, effective properties of lattice metamaterials with relative densities lower than ${\rho^{\prime }}_{min}$ were penalised to a small positive value (instead of zero, to avoid singularity) by adopting a threshold function, written as:

(16) $$\;Th\left(\tilde{\rho^{\prime }}\right)=0.5\;\left(1+\tanh\left(a\left(\tilde{\rho^{\prime }}-b\right)\right)\right)$$

where a and b are constants controlling the slope and the offset of the threshold function, respectively. With the design variable filter and threshold function applied, the effective elasticity tensor, the relative density for calculating the volume fraction objective function, and the effective yield stress can be re-written as:

(17a) $${\hat{C}}_{\zeta }^{E}\left({\tilde{\rho^{\prime }}}_{\zeta }\right)=C_{\zeta }^{E}\left({\tilde{\rho^{\prime }}}_{\zeta }\right)\cdot Th\left({\tilde{\rho^{\prime }}}_{\zeta }\right)$$

(17b) $${\hat{\rho^{\prime }}}_{\zeta }\left({\tilde{\rho^{\prime }}}_{\zeta }\right)={\tilde{\rho^{\prime }}}_{\zeta }\cdot Th\left({\tilde{\rho^{\prime }}}_{\zeta }\right)$$

(17c) $$\;{\hat{\sigma }}_{Y}^{E\zeta }\left({\tilde{\rho^{\prime }}}_{\zeta }\right)=\sigma_{Y}^{E\zeta }\left({\tilde{\rho^{\prime }}}_{\zeta }\right)\cdot Th\left({\tilde{\rho^{\prime }}}_{\zeta }\right)$$

The effective elasticity tensor with the design variable filter and the threshold function applied was adopted to calculate the stress tensor in the yield stress constraint. The stress tensor can be written as:

(18) $$\sigma_{\zeta }\left({\tilde{\rho^{\prime }}}_{\zeta }\right)={\hat{C}}_{\zeta }^{E}\left({\tilde{\rho^{\prime }}}_{\zeta }\right)B_{\zeta }U\left({\hat{C}}_{\zeta }^{E}\left({\tilde{\rho^{\prime }}}_{\zeta }\right)\right)$$

where the displacement tensor **U** is a function of ${\hat{C}}_{\zeta }^{E}\left({\tilde{\rho^{\prime }}}_{\zeta }\right)$, i.e., $U=K{\left({\hat{C}}_{\zeta }^{E}\right)}^{-1}F$, according to the equilibrium equality constraint, $h\left({\tilde{\rho^{\prime }}}_{\zeta }\right)$.

The maximum value of $\sigma_{VM}^{\zeta }/{\hat{\sigma }}_{Y}^{E\zeta }$ in the yield stress constraint was computed by using the built-in maximisation function in Nastran. To be solved in gradient-based methods, the optimisation problem should be formulated in a differentiable way. A differentiable maximisation function can be written in a p-norm form:

(19) $$\;\max\left(\sigma_{VM}^{\zeta }/{\hat{\sigma }}_{Y}^{E\zeta }\right)=\|\sigma_{VM}^{\zeta }/{\hat{\sigma }}_{Y}^{E\zeta }\|{}_{\infty }=\underset{pn\to \infty }{\lim}(\sum_{\zeta =1}^{N}{\left|\sigma_{VM}^{\zeta }/{\hat{\sigma }}_{Y}^{E\zeta }\right|}^{pn}{)}^{\frac{1}{pn}}$$

where *pn* is the exponent of the p-norm of $\sigma_{VM}^{\zeta }/{\hat{\sigma }}_{Y}^{E\zeta }$. For $\sigma_{VM}^{\zeta }/{\hat{\sigma }}_{Y}^{E\zeta }\geq 0$, $\left|\sigma_{VM}^{\zeta }/{\hat{\sigma }}_{Y}^{E\zeta }\right|=\sigma_{VM}^{\zeta }/{\hat{\sigma }}_{Y}^{E\zeta }$. Taking sufficiently large *pn*, the inequality constraint can be written as:

(20) $$\;\;g\left({\tilde{\rho^{\prime }}}_{\zeta }\right)=(\sum_{\zeta =1}^{N}(\sigma_{VM}^{\zeta }\left({\tilde{\rho^{\prime }}}_{\zeta }\right)/{\hat{\sigma }}_{Y}^{E\zeta }\left({\tilde{\rho^{\prime }}}_{\zeta }\right){)}^{pn}{)}^{\frac{1}{pn}}-c_{s}\leq 0$$

Hence, the optimisation problem ${\mathbb{P}}_{A}$ can be re-written as:

(21) $${\mathbb{P}}_{A}\left\{\begin{array}{l} \underset{{\rho^{\prime }}_{\zeta }}{\min}\;\;f\left({\rho^{\prime }}_{\zeta }\right)=0.5\;F^{T}U,\;\;\zeta =1,\ldots ,N\\ s.t.\;\;\;h\left({\rho^{\prime }}_{\zeta }\right)=K\left({\hat{C}}_{\zeta }^{E}\left({\tilde{\rho^{\prime }}}_{\zeta }\left({\rho^{\prime }}_{\gamma }\right)\right)\right)U-F=0\\ \;\;g_{1}\left({\rho^{\prime }}_{\zeta }\right)=\frac{1}{V}\sum_{\zeta =1}^{N}{\hat{\rho^{\prime }}}_{\zeta }\left({\tilde{\rho^{\prime }}}_{\zeta }\left({\rho^{\prime }}_{\gamma }\right)\right)V_{\zeta }-v_{f}\leq 0\\ \;\;g_{2}\left({\rho^{\prime }}_{\zeta }\right)=(\sum_{\zeta =1}^{N}(\frac{\sigma_{VM}^{\zeta }\left({\tilde{\rho^{\prime }}}_{\zeta }\left({\rho^{\prime }}_{\gamma }\right)\right)}{{\hat{\sigma }}_{Y}^{E\zeta }\left({\tilde{\rho^{\prime }}}_{\zeta }\left({\rho^{\prime }}_{\gamma }\right)\right)}{)}^{pn}{)}^{\frac{1}{pn}}-c_{s}\leq 0\\ \;\;{\rho^{\prime }}_{min}\leq {\rho^{\prime }}_{\zeta }\leq {\rho^{\prime }}_{max} \end{array}\right.$$

Problem ${\mathbb{P}}_{B}$ was a structural volume fraction minimisation problem under one inequality constraint, i.e., the yield stress constraint. Problem ${\mathbb{P}}_{B}$ was the simplest optimisation problem used to compare the weight reduction performance of the BCC and the BCC-F lattice structures under the yield stress constraint. ${\mathbb{P}}_{B}$ can be written as:

(22) $${\mathbb{P}}_{B}\left\{\begin{array}{l} \underset{{\rho^{\prime }}_{\zeta }}{\min}\;f\left({\rho^{\prime }}_{\zeta }\right)=\frac{1}{V}\sum_{\zeta =1}^{N}{\hat{\rho^{\prime }}}_{\zeta }\left({\tilde{\rho^{\prime }}}_{\zeta }\left({\rho^{\prime }}_{\gamma }\right)\right)V_{\zeta },\;\;\zeta =1,\ldots ,N\\ s.t.\;\;h\left({\rho^{\prime }}_{\zeta }\right)=K\left({\hat{C}}_{\zeta }^{E}\left({\tilde{\rho^{\prime }}}_{\zeta }\left({\rho^{\prime }}_{\gamma }\right)\right)\right)U-F=0\\ \;\;g\left({\rho^{\prime }}_{\zeta }\right)=\sum_{\zeta =1}^{N}(\frac{\sigma_{VM}^{\zeta }\left({\tilde{\rho^{\prime }}}_{\zeta }\left({\rho^{\prime }}_{\gamma }\right)\right)}{{\hat{\sigma }}_{Y}^{E\zeta }\left({\tilde{\rho^{\prime }}}_{\zeta }\left({\rho^{\prime }}_{\gamma }\right)\right)}{)}^{pn}{)}^{\frac{1}{pn}}-c_{s}\leq 0\\ \;\;{\rho^{\prime }}_{min}\leq {\rho^{\prime }}_{\zeta }\leq {\rho^{\prime }}_{max} \end{array}\right.$$

#### 3.3.3. Implementation of Optimisation

**Figure 13:**
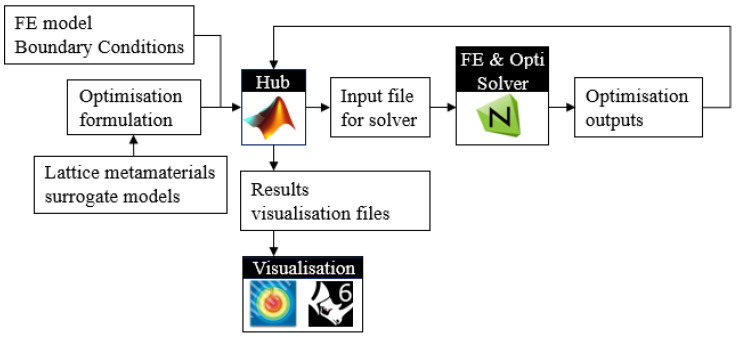
Flowchart of the optimisation platform.

An optimisation platform was developed to implement the optimisation of FGLSs. A flowchart of this platform is demonstrated in Figure 13. A MATAB program was developed as a data hub, where FE models, boundary conditions, and optimisation problem formulations were defined and written into input files for the FE analysis and optimisation solver, i.e., Nastran 2018. The optimisation problems were solved in Nastran 2018 and the optimisation output files were read and processed in the MATLAB program to generate files for visualising the optimisation results in Tecplot 2018 (contour plots) and Rhino 6 (detailed CAD models for FGLSs).

### 3.4. Optimisation Case Study

Two case studies are presented in this section to test the capability of the proposed yield constrained lattice structural optimisation framework. The first case study was an L-shaped beam bending case, in which the optimisation problem ${\mathbb{P}}_{A}$ was implemented to compare the von Mises stress of the optimised BCC-F lattice structures with and without yield stress constraint. The second case study was a Messerschmitt-Bölkow-Blohm (MBB) beam bending case, in which the optimisation problem ${\mathbb{P}}_{B}$ was implemented to compare the weight reduction capability of BCC and BCC-F lattice structures under the yield stress constraint. The safety factor, $c_{s}$, was set as one in both case studies.

As mentioned in the previous section, the FE analysis and sensitivity analysis of optimisation problems were carried out in Nastran 2018. The sensitivity analysis was conducted using the modified method of feasible directions (MMFD) [47,48]. MMFD is a built-in optimisation algorithm that is capable of efficiently solving large scale optimisation problems. In Nastran, a lattice structure was discretised into uniform sized hexahedron elements with the effective material properties of lattice metamaterials assigned to each element, so that one element corresponded to one lattice cell.

#### 3.4.1. L-Shaped Beam Case Study with Optimisation Problem ${\mathbb{P}}_{A}$

**Figure 14:**
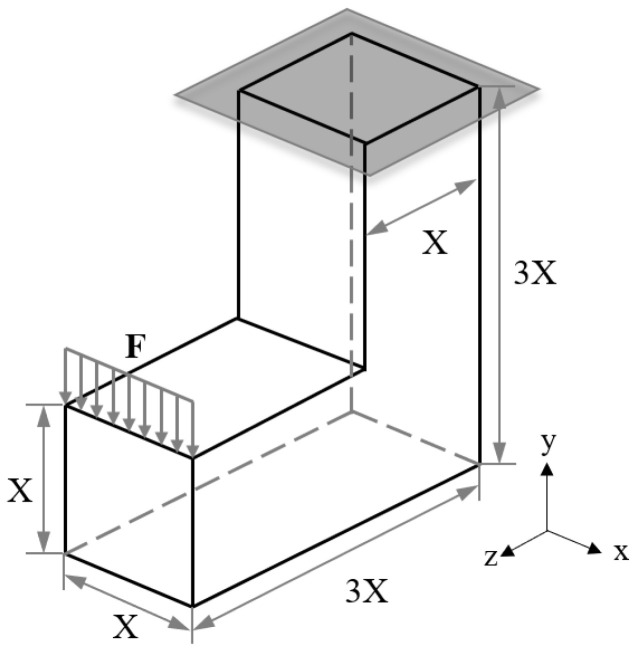
Dimensions and boundary conditions sketch of the L-shaped beam bending case study [49].

The dimensions and boundary conditions of the L-shaped beam case study are demonstrated in Figure 14. The top of the vertical part of the beam was fixed and a uniformly distributed force was applied on the top left edge of the horizontal part of the beam. The L-shaped beam was discretised into 2560 uniform sized hexahedron elements. The maximum volume fraction constraint in this case study was set to be $v_{f}$ = 0.25.

**Figure 15:**
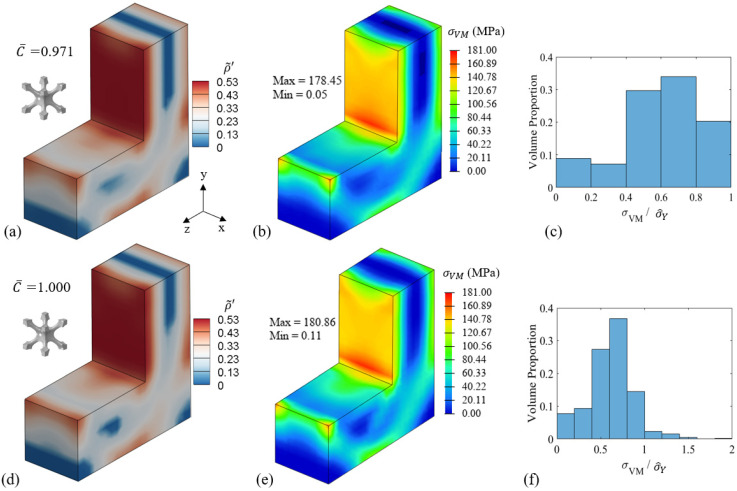
Optimisation results of the BCC-F L-shaped beam bending case study with problem ${\mathbb{P}}_{A}\left(v_{f}=0.25\right)$: distributions of (**a**) optimised relative density ($\bar{C}$ is normalised optimised compliance) and (**b**) von Mises stress, and (**c**) the volume proportion histogram of stress constraint measurement ($\sigma_{VM}/{\hat{\sigma }}_{Y}$ ) with the yield stress constraint applied; distributions of (**d**) optimised relative density and (**e**) von Mises stress, and (**f**) the volume proportion histogram of $\sigma_{VM}/{\hat{\sigma }}_{Y}$ without yield stress constraint.

The optimisation results of the distributions of relative density and von Mises stress of the BCC-F L-shaped beam are shown in Figure 15. The left column and the right column of Figure 15 show the optimisation results with and without the yield stress constraint applied, respectively. As shown in Figure 15a,d, both cases exhibited similar relative density distributions that form two hook-like higher hierarchical level structures. In both cases, the relative densities were near the upper bound in the front and the back regions of the vertical part of L-shaped beams, where the beams sustained high normal stress due to the applied bending force, as shown in Figure 15b,e.

The volume proportion histogram of stress constraint measurement ($\sigma_{VM}/{\hat{\sigma }}_{Y}$) for the two cases with and without the yield stress constraint are shown in Figure 15c,f, respectively. The value of $\sigma_{VM}/{\hat{\sigma }}_{Y}$ can be used to evaluate the safety of a lattice structure, i.e., the lattice metamaterial yields when $\sigma_{VM}/{\hat{\sigma }}_{Y}$ > 1. With the yield stress constraint applied, the value of $\sigma_{VM}/{\hat{\sigma }}_{Y}$ can be controlled below one, and thereby, the structural safety can be ensured. However, without yield stress constraint, the value of $\sigma_{VM}/{\hat{\sigma }}_{Y}$ can exceed one, indicating yield can happen in the optimised lattice structure under the applied loading conditions. For easier comparison, the values of optimised compliance of both cases are normalised by the optimised compliance of the L-shaped beam without yield stress constraint. The normalised optimised compliance ($\bar{C}$) and the maximum value of the von Mises stress for the yield stress constraint applied case were both slightly smaller than those of the case without yield stress constraint. This could be due to the fact that the yield stress constraint could guide the optimisation searching direction to optimise the stress distribution to reduce the values of $\sigma_{VM}/{\hat{\sigma }}_{Y}$ and could consequently contribute to circumventing some local optima with high values of $\sigma_{VM}/{\hat{\sigma }}_{Y}$.

**Figure 16:**
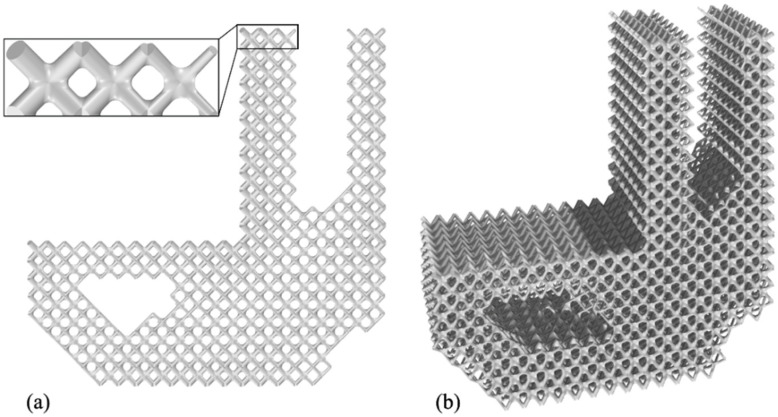
Detailed CAD model of the optimised BCC-F L-shaped beam with (**a**) front view and (**b**) perspective view.

The detailed CAD model of the optimised BCC-F L-shaped beam corresponding to Figure 15a, as shown in Figure 16, was generated from our optimisation platform presented in Section 3.3.3 using Rhino 6. It can be observed that inefficient lattice cells were completely penalised to create voids.

#### 3.4.2. MBB Beam Case Study with Optimisation Problem ${\mathbb{P}}_{B}$

**Figure 17:**
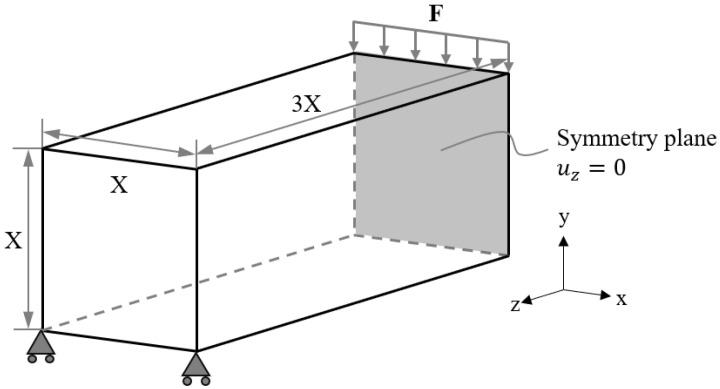
Dimensions and boundary conditions sketch of the MBB beam (half model with the symmetric boundary condition applied) bending case study.

The dimensions and the boundary conditions of the MBB beam case study are demonstrated in Figure 17. Here, only half of the beam model was computed with the symmetric boundary condition applied to the symmetry plane of the beam. The half beam model was discretised into 8 × 8 × 24 hexahedron elements with uniform element size. The optimisation problem ${\mathbb{P}}_{B}$ was implemented in this case study to compare the weight reduction capability of lattice structures with BCC and BCC-F lattice metamaterials.

**Figure 18:**
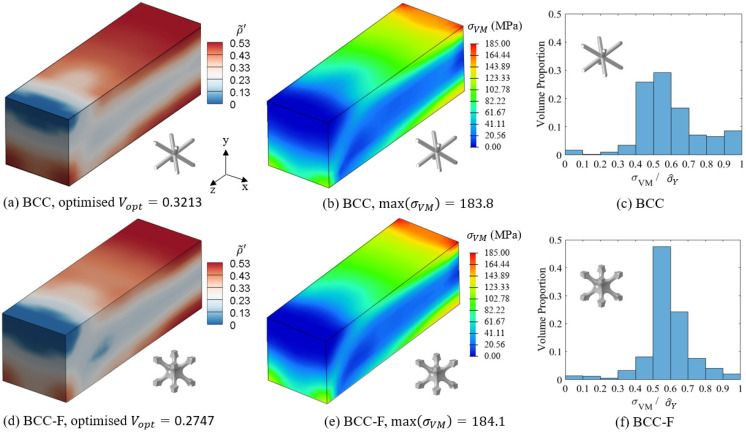
Comparisons of (**a**,**d**) the relative density (${\tilde{\rho }}^{\prime }$) distributions and the optimised volume fractions ($V_{opt}$ ), (**b**,**e**) the von Mises stress ($\sigma_{VM}$ ) contours, and (**c**,**f**) the volume proportion histogram of $\sigma_{VM}/{\hat{\sigma }}_{Y}$ for the optimised MBB beam with the BCC (the top row) and the BCC-F (the bottom row) lattices.

The distributions of the relative density and the von Mises stress of the optimised BCC and BCC-F lattice structures are compared in Figure 18. Similar bridge-like relative density distributions can be observed in both BCC and BCC-F lattice structures. Lattice metamaterials with high relative densities distributed in the top and bottom regions of the beams near the symmetry surface where the beams were subject to high normal stress cause by the bending force and the left bottom edge of the beams where boundary conditions were applied. The regions between the top and bottom high relative density regions of the beams were filled lattice metamaterials with moderate relative densities. The distributions of the high relative density regions in the optimised BCC and BCC-F lattice structures were similar. However, more low relative density lattice metamaterials were observed in the moderate relative density region in the BCC-F lattice structure compared to the BCC lattice structure.

The optimisation results showed that the BCC-F lattice structure achieved 0.0466 more reduction in the optimised volume fraction, $V_{opt}$, when compared with the BCC lattice structure, which is a 14.5% improvement. The maximum von Mises stress of the optimised BCC-F lattice structure was only slightly higher than that of the optimised BCC lattice structure, without exceeding their yield criteria for both cases. It indicates that adding fillets to the joints of the BCC type of lattice metamaterials can contribute to higher structural efficiency by increasing the yield stress of the lattice metamaterials.

The histogram plots of the volume proportion distribution of $\sigma_{VM}/{\hat{\sigma }}_{Y}$ for the optimised BCC and BCC-F lattice structures are shown in Figure 18c,f, respectively. The values of $\sigma_{VM}/{\hat{\sigma }}_{Y}$ for both BCC and BCC-F lattice structures were successfully controlled, i.e., they were not exceeding one, indicating the structural safety of both lattice structures was ensured. It can also be observed that the proportion of high $\sigma_{VM}/{\hat{\sigma }}_{Y}$ values, i.e., $\sigma_{VM}/{\hat{\sigma }}_{Y}$ > 0.8, in the optimised BCC-F lattice MBB beam was lower than that in the optimised BCC lattice MBB beam. More specifically, there were only 6.0% of lattice metamaterials subject to $\sigma_{VM}/{\hat{\sigma }}_{Y}$ > 0.8 in the optimised BCC-F lattice MBB beam, while the proportion was 15.1% for the optimised BCC lattice MBB beam. It indicates that introducing fillets to the BCC type lattice metamaterials can improve the structural safety of the optimised lattice structure.

## 4. Conclusions

A framework for optimising the structural performance of functionally graded lattice structures (FGLSs) has been developed in this paper, with a particular focus on enhancing and guaranteeing structural safety through three contributions: firstly, from the lattice cell design perspective, new fillet features have been added to the joints of BCC lattice cells to reduce stress concentration; secondly, from the lattice metamaterials characterisation perspective, the effective material properties (effective elasticity tensors and effective yield stresses) of BCC and filleted BCC (BCC-F) lattices have been experimentally investigated and characterised to mitigate the discrepancies between the numerically characterised and the actual effective material properties; thirdly, a von Mises yield stress constraint for the optimisation of FGLSs has been developed based on the surrogate models of the effective material properties of the BCC and BCC-F lattices, developed using the experimental results with the assistance of numerical homogenisation simulations. The proposed framework has been implemented by establishing an optimisation platform based on MATLAB and Nastran and tested through an L-shaped beam bending case study and an MBB beam bending case study.

The key findings of this paper are summarised as follows: (i) The effective yield stress of the BCC type lattice metamaterials increases monotonically with the increase of fillet parameter, $N_{f}$ (see Equation (2)), within the given range (1 to 10) of $N_{f}$, and the improvement in effective yield stress becomes less significant with increasing relative density of the lattice metamaterials; (ii) The experimentally characterised effective Young’s moduli and yield stresses of both lattices are lower than the corresponding numerical homogenisation results and the discrepancies increase with the decrease of the relative densities of the lattice metamaterials; (iii) Imposing the developed yield stress constraint can successfully ensure the structural safety of the optimised beams in both case studies. Additionally, introducing fillets to the strut joints of the BCC type lattices can effectively improve the lightweighting capability and structural safety of the optimised lattice structures. In the MBB beam case study, the maximum von Mises stresses in the optimised beams are successfully controlled below yield stresses, and the optimised BCC-F lattice beam achieved 14.5% improvement in weight reduction compared with the optimised conventional BCC lattice beam.

To broaden the applications in the future, the framework developed in this paper can be extended to include a wide range of bending dominated lattice metamaterials, such as primitive cubic lattices.

## Figures and Tables

**Table 1:** Abbreviations used in the paper.

Abbreviation	Description
FGLS	Functionally graded lattice structures
BCC	Body-centred cubic
BCC-F	Filleted body-centred cubic
AM	Additive manufacturing
SLM	Selective laser melting
DMLS	Direct metal laser sintering
PC	Primitive cubic
FE	Finite element
RVE	Representative volume element
PBC	Periodic boundary condition

**Table 2:** Material and processing parameters of SLM.

SLM Processing Parameters	Values
Material	Ti6Al4V
Powder grade	Gd5
Spherical particle size (μm)	15–53
Laser power (W)	320
Laser scanning speed (mm/s)	1200
Hatch spacing (mm)	0.14
Layer thickness (mm)	0.04

## Data Availability

The data presented in this study are available upon reasonable request from the corresponding author.
